# Individual-level home values and cancer mortality in a statewide registry

**DOI:** 10.1093/jncics/pkad076

**Published:** 2023-10-05

**Authors:** Alec Zhu, Stephen Rhodes, Weichuan Dong, Johnie Rose, Jennifer Cullen, David B Miller, Daniel E Spratt, Lee Ponsky, Daniel Shoag, Erika Trapl, Fredrick Schumacher, Suhas Penukonda, Aaron Brant, Mary O Strasser, Siran M Koroukian, Sarah Markt, Jonathan E Shoag

**Affiliations:** Department of Urology, New York-Presbyterian Hospital, Weill Cornell Medicine, New York, NY, USA; Department of Urology, University Hospitals Cleveland Medical Center, Case Western Reserve University School of Medicine, Cleveland, OH, USA; Department of Population and Quantitative Health Sciences, Case Western Reserve University School of Medicine, Cleveland, OH, USA; Department of Population and Quantitative Health Sciences, Case Western Reserve University School of Medicine, Cleveland, OH, USA; Department of Population and Quantitative Health Sciences, Case Western Reserve University School of Medicine, Cleveland, OH, USA; Department of Social Work, Mandel School of Applied Social Sciences, Case Western Reserve University School of Medicine, Cleveland, OH, USA; Department of Radiation Oncology, Seidman Cancer Center, University Hospitals Cleveland Medical Center, Case Western Reserve University School of Medicine, Cleveland, OH, USA; Department of Urology, University Hospitals Cleveland Medical Center, Case Western Reserve University School of Medicine, Cleveland, OH, USA; Department of Economics, Weatherhead School of Management, Case Western Reserve University School of Medicine, Cleveland, OH, USA; Department of Population and Quantitative Health Sciences, Case Western Reserve University School of Medicine, Cleveland, OH, USA; Department of Population and Quantitative Health Sciences, Case Western Reserve University School of Medicine, Cleveland, OH, USA; Department of Urology, New York-Presbyterian Hospital, Weill Cornell Medicine, New York, NY, USA; Department of Urology, New York-Presbyterian Hospital, Weill Cornell Medicine, New York, NY, USA; Department of Urology, New York-Presbyterian Hospital, Weill Cornell Medicine, New York, NY, USA; Department of Population and Quantitative Health Sciences, Case Western Reserve University School of Medicine, Cleveland, OH, USA; Department of Population and Quantitative Health Sciences, Case Western Reserve University School of Medicine, Cleveland, OH, USA; Department of Urology, New York-Presbyterian Hospital, Weill Cornell Medicine, New York, NY, USA; Department of Urology, University Hospitals Cleveland Medical Center, Case Western Reserve University School of Medicine, Cleveland, OH, USA

## Abstract

**Background:**

Prior work assessing disparities in cancer outcomes has relied on regional socioeconomic metrics. These metrics average data across many individuals, resulting in a loss of granularity and confounding with other regional factors.

**Methods:**

Using patients’ addresses at the time of diagnosis from the Ohio Cancer Incidence Surveillance System, we retrieved individual home price estimates from an online real estate marketplace. This individual-level estimate was compared with the Area Deprivation Index (ADI) at the census block group level. Multivariable Cox proportional hazards models were used to determine the relationship between home price estimates and all-cause and cancer-specific mortality.

**Results:**

A total of 667 277 patients in Ohio Cancer Incidence Surveillance System were linked to individual home prices across 16 cancers. Increasing home prices, adjusted for age, stage at diagnosis, and ADI, were associated with a decrease in the hazard of all-cause and cancer-specific mortality (hazard ratio [HR] = 0.92, 95% confidence interval [CI] = 0.92 to 0.93, and HR = 0.95, 95% CI = 0.94 to 0.95, respectively). Following a cancer diagnosis, individuals with home prices 2 standard deviations above the mean had an estimated 10-year survival probability (7.8%, 95% CI = 7.2% to 8.3%) higher than those with home prices 2 standard deviations below the mean. The association between home price and mortality was substantially more prominent for patients living in less deprived census block groups (*P*_interaction_ < .001) than for those living in more deprived census block groups.

**Conclusion:**

Higher individual home prices were associated with improved all-cause and cancer-specific mortality, even after accounting for regional measures of deprivation.

Few studies have been able to assess individual-level income associations with health outcomes. A study from the United States found that women and men in the top 1% of household income lived 10 and 15 years longer, respectively, than their respective counterparts in the bottom 1% (1). However, assessing the relationship between cancer outcomes and individual-level metrics of income in the United States has not previously been feasible. As such, prior work has primarily relied on regional metrics, which, at the most granular level, average data across census block groups, which comprise between 600 and 3000 individuals (2-4). Averaging across hundreds or thousands of individuals, although necessary given the limitations of most datasets, loses granularity and confounds the impact of income with other regional factors (5). Given the close relationship between income and house prices (6,7) as well as the limited availability of individual-level income data, this study evaluated whether estimated home prices, as a proxy for individual income, are associated with survival following a cancer diagnosis for patients within a statewide cancer registry (8).

## Methods

### Study design and population

This retrospective study examined patients captured within the Ohio Cancer Incidence Surveillance System (OCISS)—a population-based registry that includes cancer incidence and longitudinal outcomes data for all residents of Ohio (8). Cancers with the highest 2022 estimated incidences and/or mortalities were selected (9). Patients aged 18 years and older and diagnosed with one of the following cancers, between 1996 and 2016, were included: bladder, brain, breast, colon or rectum, esophagus, kidney, leukemia, liver, lung, lymphoma, melanoma, ovarian, pancreatic, prostate, stomach, or uterine cancer. Follow-up extended through 2018. Demographic data, stage at diagnosis, and year of diagnosis were captured. Cancer sites were identified based on the International Classification of Diseases for Oncology Third Edition codes (Supplementary Table 1, available online). Stage at diagnosis was separated into categories of in situ, localized, regional, distant, or unstaged for most cancers. The in situ categorization was excluded for cancers that do not utilize this form of staging. Only distant stage at diagnosis was used for leukemia (10). Number of days from diagnosis to all-cause or cancer-specific mortality was the primary outcomes of interest. Cancer-specific mortality was determined by death certificate reports, and the “Surv-Date Presumed Alive” variable in OCISS was used to determine the date of death or censoring. This study was approved by the Ohio Department of Health (protocol #2019-5) and the Case Western Reserve University (protocol #20190455) institutional review boards.

### Income measurement

Individual home addresses, obtained from the OCISS, were linked to home price estimates (Zestimate) from Zillow, which is a publicly available tool. Although the method of calculating Zestimate is proprietary, the Zestimate utilizes public data, such as home details, location, property tax assessments, sales histories, and other homes recently sold in the area (11). We used home price estimates in 2022 because historical estimates are not available. Although home prices have risen and market fluctuations have occurred recently because of the global pandemic (12), we assumed the relative rank ordering of home prices was stable over time (13). As missing data are likely nonrandom, we analyzed differences between linked and unlinked participants.

Individual data were also linked to census block group-level data to estimate neighborhood deprivation using the Area Deprivation Index (ADI), which was obtained from the 2019 American Community Survey 5-year estimates. The ADI is a measure of economic and material deprivation consisting of 15 US census variables, such as median family income, education distribution, and unemployment rate, and is calculated at the census block level (3,14).

### Statistical analysis

Survival was the outcome of choice as it is readily available in OCISS, reflects variability in all stages of cancer care (screening, diagnosis, treatment), and is generally of most interest to patients following a cancer diagnosis (15). Survival times for all-cause or cancer-specific mortality were analyzed via Cox proportional hazards models. Home price was log transformed, *z* scored, and included as a linear predictor. Age at diagnosis was modeled via a restricted cubic spline with 4 knots. ADI was also *z* scored for analysis and included as a continuous variable in the Cox models.

To assess the strength of the association between home prices and survival, unadjusted and adjusted Cox models were constructed. The hazard ratio (HR) per standard deviation change in home price was assessed, adjusting for covariates of age at diagnosis, stage at diagnosis, and/or ADI. A conceptual model used to direct our analyses demonstrates income, or home price as a proxy, is assumed to influence survival through various mediators including quality-of-care, age, and stage at diagnosis (Supplementary Figure 1, available online). Although quality-of-care data are not available, adjusting for age and stage estimates the influence of income on survival.

Cox models were generated for each cancer individually and for all cancers combined; unadjusted and adjusted analyses of all cancers combined incorporated cancer site as a covariate. These models were used to calculate marginal 5- and 10-year survival probabilities at different home price estimates using the Kalbfleisch–Prentice estimate of the survival function (16). Percentile confidence intervals (CIs) for survival probabilities were calculated via bootstrapping with 1000 replicates.

Because area deprivation may also influence quality-of-care and survival outcomes (Supplementary Figure 1, available online), we generated a model including age, stage at diagnosis, home price, ADI, and the interaction between home price and ADI to assess how the relationship between home price and mortality varies by ADI. Additionally, Kaplan–Meier tables of median survival times, stratified by quartiles of ADI and home price, were generated for all cancers.

To compare the utility of individual home price as a measure of economic resources with ADI, separate Cox models were fit including either home price or ADI as predictors alone or controlling for age and stage. The hazard ratios per 1 standard deviation change in home price or ADI were compared, and predictive utility was assessed via the Akaike Information Criterion (AIC).

As home values were collected in 2022 for all patients, additional analysis was performed to determine the impact of year of diagnosis on the relationship between home price and mortality. Cox models determined the relationship between hazard of mortality and home price with patients stratified by early (1996-2005) or later (2006-2016) years of cancer diagnosis.

A parallel analysis obtained an estimation of income by adjusting individual home prices by census block group estimates of the proportion of income spent on housing-related costs—obtained from the American Community Survey—for those with and without a mortgage (17). Home price was divided by the estimated median proportion of income spent on housing, which was then used for the same models to determine the relationship between income and mortality. Statistical significance was defined as a *P* value less than .05, and all tests were 2-sided. All analyses were performed in R, version 4.2.1. The sociome, rms, and survival packages were used to obtain ADI and perform survival analyses (18-20).

## Results

A total of 1 058 123 patients in OCISS were identified across the 16 cancers of interest. Of these patients, 11 332 (1.1%) individuals were excluded for being aged younger than 18 years and/or lacking cancer stage information. An additional 379 514 (36%) patients were excluded for missing home prices and/or ADI. Differences between linked and unlinked patients are shown in Supplementary Table 2 (available online).

**Figure 1.**
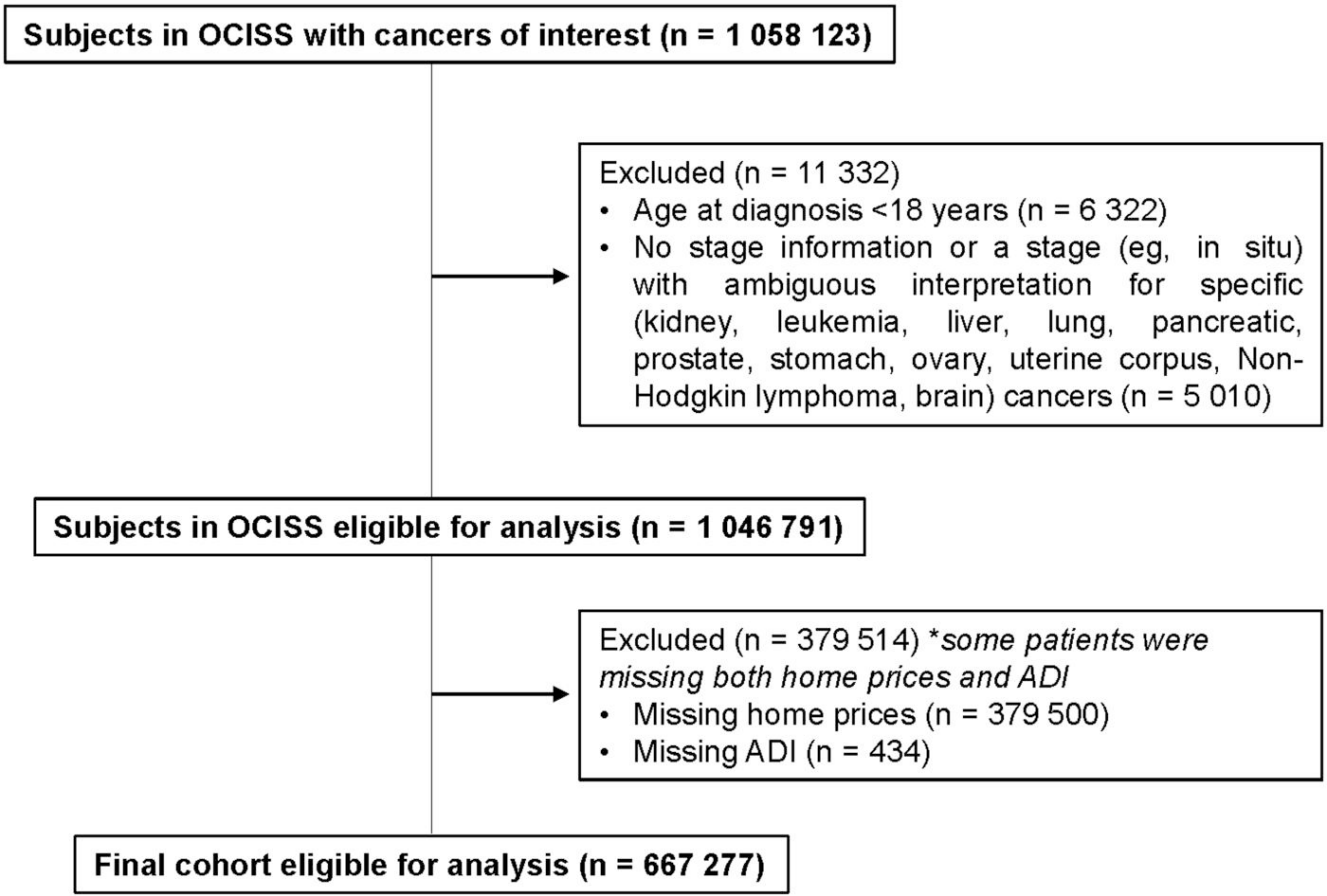
Study flow diagram. ADI = Area Deprivation Index; OCISS = Ohio Cancer Incidence Surveillance System.

The final cohort of linked patients consisted of 667 277 patients (Figure 1). Baseline demographics and mortality outcomes of patients diagnosed with each cancer are shown in Supplementary Table 3 (available online). Median home price across all malignancies was $201 500 (interquartile range [IQR] = $140 900-$291 800). Median days of follow-up across all malignancies were 1290 days (IQR = 338-3142 days).

**Figure 2.**
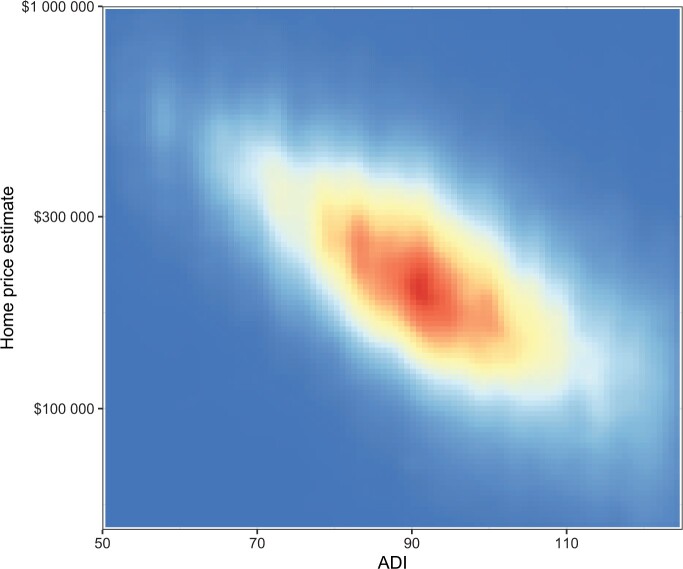
Distribution of home prices according to census block groups. Plot of individual home prices according to census block groups ranked by Area Deprivation Index (ADI). **Red and yellow** colors indicate home prices within a census block group. **Darker shades of red** indicate higher density of home prices within a particular census block group. Census block groups are ranked from lowest to highest (left to right) ADI.

As expected, patients living in areas with lower ADI (low deprivation) generally had higher home values compared with patients living in areas with higher ADI (high deprivation). However, there was wide variation in estimated home values across census block groups with different ADI values (Figure 2).

**Figure 3.**
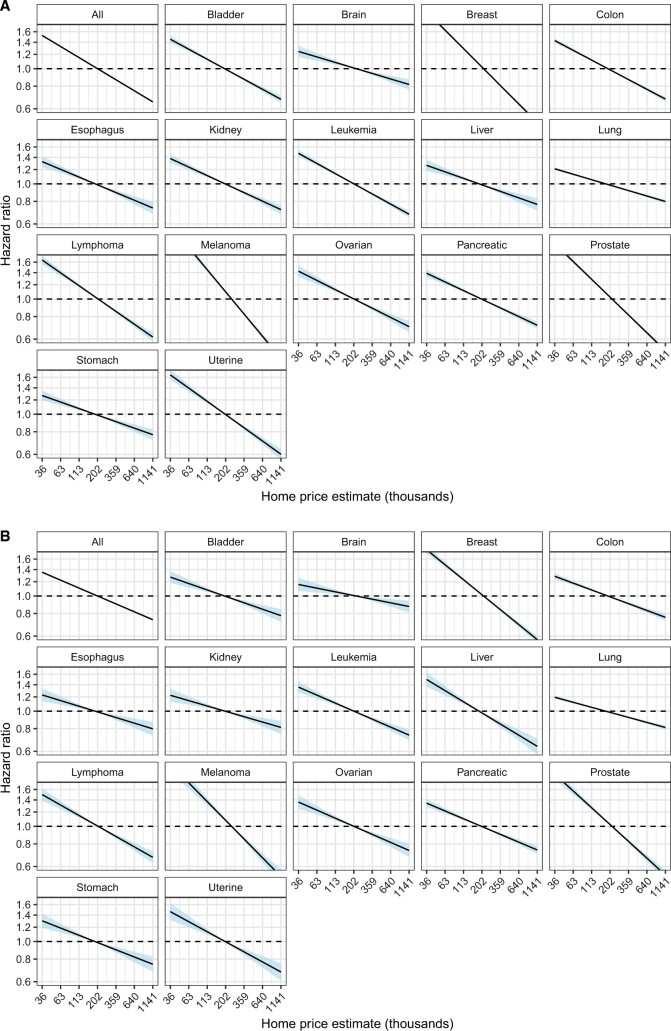
Overall relationship between hazard of mortality and home price. Unadjusted Cox models assessing the relationship between hazard of all-cause **(A)** and cancer-specific **(B)** mortality and home price for cancers of all stages combined. Home prices were log transformed and *z* scored and scaled. The model for all cancers combined included cancer site as a covariate. Shading indicates 95% confidence interval bands.

Cox models assessing the relationship between home prices and mortality (Figure 3) found that increasing home prices were associated with a lower hazard of all-cause mortality across all cancers in both an unadjusted model (HR = 0.87, 95% CI = 0.87 to 0.87 per standard deviation increase in log home price; Supplementary Table 4, available online) and a model adjusted for age and stage at diagnosis (HR = 0.89, 95% CI = 0.89 to 0.89; Supplementary Table 5, available online). Additionally, increasing home prices were associated with a lower hazard of cancer-specific mortality across all cancers in an unadjusted model (HR = 0.90, 95% CI = 0.90 to 0.91) and a model adjusted for age and stage (HR = 0.92, 95% CI = 0.91 to 0.92). Home prices were also associated with all-cause and cancer-specific mortality in cancers stratified by stage at diagnosis. (Supplementary Figure 2, available online). In models incorporating age, stage at diagnosis, cancer site, and ADI, increasing home price was associated with diminished all-cause (HR = 0.92, 95% CI = 0.92 to 0.93) and cancer-specific (HR = 0.95, 95% CI = 0.94 to 0.95) mortality for all cancers combined (Table 1). When stratified by cancer type, the association between home prices and all-cause mortality was statistically significant for all 16 cancers and the association between home prices and cancer-specific mortality was statistically significant for 11 of 16 cancers in models adjusting for age, stage, and ADI (Table 1). Estimated all-cause 5-year survival probabilities, generated from Cox models incorporating home price, cancer type, age, and ADI, were greater for individuals with home prices at 2 standard deviations above the mean home price compared with those with home prices 2 standard deviations below the mean (62.7% vs 55.7%; Supplementary Table 6, available online), with a difference of 7.0% (95% CI = 6.5% to 7.4%). At 10 years, the difference in probability of survival between those with high and low home prices is 7.8% (95% CI = 7.2% to 8.3%).

**Table 1.** Hazard ratios from adjusted Cox proportional hazards models for all-cause and cancer-specific mortality based on home price, age at diagnosis, stage at diagnosis, and Area Deprivation Index for all cancers combined and each cancer individually^a^

Cancer Type	All-cause mortality	Cancer-specific mortality
	HR (95% CI)	HR (95% CI)
All cancers	0.924 (0.920 to 0.928)	0.949 (0.943 to 0.954)
Bladder	0.930 (0.911 to 0.948)	0.974 (0.941 to 1.007)
Brain	0.941 (0.912 to 0.971)	0.961 (0.927 to 0.996)
Breast	0.936 (0.923 to 0.948)	0.953 (0.933 to 0.973)
Colon or rectum	0.946 (0.934 to 0.958)	0.972 (0.956 to 0.989)
Esophagus	0.952 (0.922 to 0.984)	0.973 (0.937 to 1.010)
Kidney	0.951 (0.927 to 0.975)	0.986 (0.951 to 1.022)
Leukemia	0.926 (0.909 to 0.943)	0.934 (0.906 to 0.962)
Liver	0.942 (0.911 to 0.975)	0.892 (0.854 to 0.933)
Lung	0.945 (0.936 to 0.953)	0.949 (0.940 to 0.959)
Lymphoma	0.916 (0.894 to 0.939)	0.933 (0.901 to 0.966)
Melanoma	0.886 (0.866 to 0.906)	0.927 (0.886 to 0.969)
Ovarian	0.950 (0.919 to 0.981)	0.950 (0.915 to 0.987)
Pancreatic	0.931 (0.912 to 0.951)	0.947 (0.926 to 0.969)
Prostate	0.894 (0.882 to 0.907)	0.939 (0.912 to 0.966)
Stomach	0.940 (0.912 to 0.968)	0.961 (0.921 to 1.003)
Uterine	0.923 (0.896 to 0.951)	0.982 (0.936 to 1.029)

a CI = confidence interval; HR = hazard ratio.

**Figure 4.**
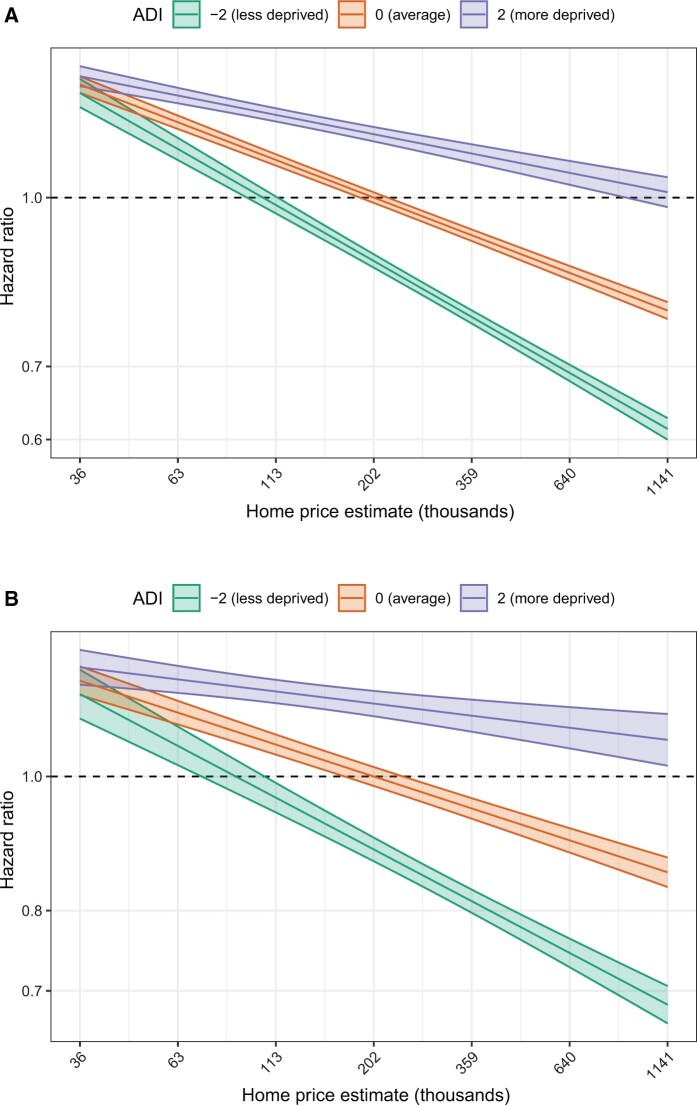
Effect of area deprivation and home price on hazard of mortality. Hazard ratios from Cox models allowed for an interaction between home price and Area Deprivation Index (ADI) for all-cause **(A)** and cancer-specific **(B)** mortality in all cancers combined. Hazard ratios are plotted for ADI values 2 standard deviations above (**blue**) or below (**green**) the mean (**red**). Cox models were constructed with ADI as a continuous (linear) predictor along with home price, age at diagnosis, stage at diagnosis, and cancer site. Hazard ratios are given at the average age and localized stage at diagnosis and have been rescaled to make home price equal to zero and ADI equal to zero the referent. Outer borders of each line represent 95% confidence interval bands.

The discriminative abilities of individual home price and ADI were compared across all cancers with and without adjustment for age and stage at diagnosis. We focused on absolute differences in an AIC of at least 10 as this typically represents strong evidence in favor of one model over another (21). According to the AIC, unadjusted Cox models utilizing home price for all-cause mortality had stronger predictive value compared with models using ADI for all cancers combined (ΔAIC > 1955.91) and for 15 of 16 cancers when assessed individually (ΔAIC > 10); models using home price remained superior for 10 of 16 cancers when adjusting for age or when adjusting for age and stage at diagnosis (Table 2). Additionally, models utilizing both home price and ADI tended to have stronger predictive ability than models using only home price (ΔAIC > 403.19) or ADI (ΔAIC > 2359.09) for all cancers combined (Table 3). The change in hazard ratio per 1 standard deviation change in home price was typically larger than the change in hazard ratio per standard deviation change in ADI (Supplementary Figure 3, available online). Models assessing the interaction between home price and ADI identified a statistically significant interaction between ADI and home price in predicting all-cause and cancer-specific mortality for all cancers combined (both *P*_interaction_  < .001 for term; Supplementary Table 7, available online). The relationship between home price and the hazard of mortality was greater for patients living in less deprived regions (Figure 4). The results for each cancer analyzed separately are shown in Supplementary Figure 4 (available online).

**Table 2.** Difference in Akaike Information Criterion assessing the relative quality of models using home price compared with models using Area Deprivation Index to predict all-cause or cancer-specific mortality, with or without adjustment for age and stage at diagnosis, for all cancers combined and each cancer individually^a^

Adjustment for	All-cause mortality			Cancer-specific mortality		
	None	Age	Age and stage	None	Age	Age and stage
All cancers	−1955.91	−565.48	−494.97	−258.48	11.98	21.04
Bladder	−73.52	−5.6	−22.69	3.6	14.82	2.88
Brain	−28.29	−16.97	−14.37	−10.25	−5.83	−4.74
Breast	−542.63	71.07	95.95	6.85	60.36	78.38
Colon or rectum	−167.47	−15.9	−11.57	26.32	44.89	33.4
Esophagus	−0.74	7.71	2.2	4.59	8.64	3.74
Kidney	−47.88	−10.21	15.09	−7.75	0.2	12.04
Leukemia	−106.59	−42.4	−42.4	−34.61	−17.86	−17.86
Liver	−21.9	−13.54	−5.08	−34.94	−29.29	−21.69
Lung	−75.64	−41.23	−74.73	−45.04	−26	−50.12
Lymphoma	−111.51	−21.08	−16.41	−38.32	−6.87	−5.22
Melanoma	−312.91	−70.57	−63.4	−18.87	−8.9	−3.93
Ovarian	−21.62	1.72	6.2	−14.64	−1.14	0.94
Pancreatic	−63.46	−45.79	−26.48	−33.29	−19.51	−3.97
Prostate	−715.14	−211.21	−191.2	−69.39	10.68	9.15
Stomach	−23.52	−6.16	−15.36	0.17	8.52	3.57
Uterine	−26.02	8.51	6.83	21.37	29	19.64

a An absolute difference in Akaike Information Criterion of at least 10 indicates stronger predictive value of one model over another. Negative values indicate greater preference for models using home price compared with models using Area Deprivation Index.

**Table 3.** Difference in AIC assessing the relative quality of models using both home price and ADI to predict all-cause mortality compared with models using only home price or ADI for all cancers combined and each cancer individually^a^

Models with home price and ADI compared with models with only	All-cause mortality	
	Home price	ADI
All cancers	−403.19	−2359.09
Bladder	−19.15	−92.67
Brain	−0.83	−29.11
Breast	−154.31	−696.94
Colon or rectum	−53.07	−220.55
Esophagus	−10.65	−11.39
Kidney	−1.34	−49.21
Leukemia	−14.19	−120.78
Liver	1.97	−19.92
Lung	−46.70	−122.33
Lymphoma	−9.70	−121.21
Melanoma	−65.31	−378.22
Ovarian	−5.09	−26.70
Pancreatic	−5.43	−68.89
Prostate	−42.25	−757.39
Stomach	1.14	−22.38
Uterine	−30.17	−56.19

a An absolute difference in AIC of at least 10 indicates stronger predictive value of one model over another. Negative values indicate greater preference for models using both home price and ADI compared with models using only home price or ADI. ADI = Area Deprivation Index; AIC = Akaike Information Criterion.

As an alternative means of assessing the interaction between ADI and home price, Kaplan–Meier estimates of median survival times for all cancers stratified by quartile of home price and ADI were generated (Table 4). Within an ADI quartile, median survival times were higher at higher home price quartiles. Patients living in the highest ADI quartile (most deprived) with the lowest home prices had lower median all-cause survival times compared with patients living in the lowest ADI quartile with the highest home prices (1638 vs 5091 days). Survival times for each individual cancer are included in Supplementary Table 8 (available online).

**Table 4.** Kaplan–Meier median all-cause survival times for all cancers stratified by quartile of home price estimates and ADI^a^

Home price quartile	ADI quartile 1	ADI quartile 2	ADI quartile 3	ADI quartile 4
	Median survival (95% CI), days (Least deprived)	Median survival (95% CI), days	Median survival (95% CI), days	Median survival (95% CI), days (Most deprived)
Home price quartile 1	1969 (1806 to 2180)	2019 (1922 to 2107)	1853 (1814 to 1906)	1638 (1605 to 1667)
Home price quartile 2	2624 (2492 to 2740)	2436 (2371 to 2490)	2204 (2153 to 2253)	1986 (1922 to 2042)
Home price quartile 3	3193 (3124 to 3262)	2919 (2864 to 2978)	2764 (2682 to 2847)	2359 (2280 to 2463)
Home price quartile 4	5091 (5009 to 5166)	4183 (4056 to 4307)	3747 (3612 to 3909)	3126 (2924 to 3353)

a ADI = Area Deprivation Index; CI = confidence interval.

For sensitivity analysis, we assessed the impact of incorporating year of diagnosis, or home price adjusted for the estimated median proportion of income spent on housing-related costs, on the relationship between individual home prices and cancer outcomes. We found a similar trend of decreasing hazard of mortality with increasing home price across all cancers as well as each individual cancer regardless of earlier (1996-2005) or later (2006-2016) years of diagnosis (Supplementary Figure 5, available online). We also found that adjustment for the regional proportion of income spent on housing had a minimal impact on the relationship between individual home prices and cancer outcomes (Supplementary Tables 9-12, Supplementary Figures 6 and 7, available online).

Given a large proportion of patients with missing home prices, we assessed the relationship between ADI and all-cause mortality (adjusting for cancer, age, and stage at diagnosis) and demonstrated a similar hazard ratio in the total cohort (n = 1 046 357; HR = 1.13, 95% CI = 1.13 to 1.13) compared with the hazard ratio in the cohort with home prices available (n = 667 291; HR = 1.13, 95% CI = 1.12 to 1.13).

## Discussion

Using a statewide cancer registry, our results are the first to demonstrate that individual home value, a surrogate marker of income, is associated with all-cause and cancer-specific mortality across multiple cancers in the United States. This is consistent with prior studies that have used individual-level data and demonstrated the relationship between income and life expectancy (1,22), as well as prior work in a Danish population from 1987 to 2013, which identified better outcomes for cancer patients with higher individual incomes (23).

Many epidemiological studies have demonstrated that patients of lower socioeconomic status have worse cancer survival outcomes (24-31). For example, Singh et al. (31) demonstrated cancer survival from 1950 to 2014 was lower for individuals living in more disadvantaged areas, and these disparities remained after adjusting for stage at diagnosis. Furthermore, Afshar et al. (25) showed individuals living in more disadvantaged areas had lower 5-year survival across 21 of 29 cancer types, but one of the main limitations noted was lack of individual-level data on socioeconomic status. For our study, we used the ADI, which is perhaps the gold standard for summarizing the effects of living in a particular neighborhood (32), as it incorporates a host of regional factors including income, education, employment, and housing quality, to quantify the degree of deprivation at a census block–level (3,14). Analyses using the ADI have shown that cancer patients living in areas of high deprivation have worse clinical outcomes (33-35).

Individuals within a census block may have a range of socioeconomic statuses (4), with prior work demonstrating that there is low concordance between individual- and area-level factors of deprivation (5,36). Consistent with this variability, we found individual home prices vary substantially across all deprivation indices, and the lack of individual-level granularity in regional metrics underestimates the impact of socioeconomic status on survival outcomes. Across all cancers, increasing home values were associated with decreasing risk of all-cause and cancer-specific mortality, and this effect was more pronounced for patients living in areas of low deprivation. The interaction between home price and ADI suggests that individuals living in high deprivation areas may experience less improvement in survival outcomes as home values increase compared with individuals living in low deprivation areas, likely due to other factors, such as access to care. Furthermore, when comparing the predictive ability of home price and ADI for mortality outcomes, we demonstrate that home price has a stronger predictive ability than ADI for most cancers, and models that utilize both home price and ADI are superior to models that use only home price or ADI.

Our study must be considered within the context of its limitations. First, estimated home values may not accurately reflect true home prices sold on the market. Nevertheless, the median error rate of the Zestimate for on-market homes is 3.2% in Ohio, and 87.8% of sales had Zestimates within 10% of the transaction price (37). Home values based on Zestimates were used as a surrogate marker for individual income, but this may not completely reflect one’s overall wealth. Assets beyond home ownership, such as retirement plans, investments, and businesses, are not accounted for, and individuals who rent homes or live in rural areas where Zestimates are unavailable would have a less accurate representation of their income using our analysis. Nevertheless, home equity is the most significant contributor to individual wealth for net worths up to $1 million (38), which would encompass most individuals. Also, we adjusted home prices by the median proportion of income spent on housing-related costs (utilities, taxes, mortgages, etc.) and showed that the relationship between home price and cancer outcomes remained consistent across cancers. Another limitation of this study is the exclusion of patients based on missing (36%) home price data. Zillow does not provide estimates for rental properties and for small counties with limited transactional data (39). Patients with missing home prices lived in regions with higher ADIs (more deprived), were less likely to live in urban areas, and had a lower proportion of owner-occupied households (Supplementary Table 2, available online). Exclusion of these individuals did not significantly impact the relationship between ADI and survival and therefore is unlikely to impact the relationship between home price and survival. In fact, exclusion of patients missing home prices may lead to residual underestimation of the relationship between home price and mortality. Furthermore, home values determined by the Zestimate for all patients reflect values in 2022 and may not accurately reflect the home prices of patients when they were diagnosed with cancer. However, sensitivity analysis stratifying patients by earlier (1996-2005) or later (2006-2016) years of diagnosis demonstrated a similar trend of decreasing mortality risk with increasing home value regardless of the year of diagnosis (Supplementary Figure 5, available online). Finally, data on comorbidities, which may confound the relationship between income and survival, are unavailable in OCISS and cannot be accounted for in our analysis.

We show that higher individual home value was associated with improvement in mortality outcomes across multiple cancer types. Even controlling for area deprivation, individuals with higher home prices have higher long-term survival probabilities after cancer diagnosis. For individuals living in less-deprived (wealthier) areas, the impact of income on survival was even more prominent, whereas among individuals from more deprived (poorer) areas, there was less of an impact on survival associated with home prices. Although further work is needed, our analysis highlights dramatic disparities in outcomes associated with socioeconomic status, independent of region, and allows for the separate definition of individual and regional factors that account for disparate outcomes.

## Supplementary Material

pkad076_Supplementary_Data

## Data Availability

Patient-level data used in this study are not available, because they were obtained under license from the Ohio Cancer Incidence Surveillance System. Codes used for data processing and statistical analysis can be made available upon request to the corresponding author.
