# Supplementary material for: Individual-level home values and cancer mortality in a statewide registry
Source: JNCI Cancer Spectr. 2023 Oct 5;7(6):pkad076. doi: 10.1093/jncics/pkad076 (PMC10646779; doi:10.1093/jncics/pkad076)

SUPPLEMENTARY MATERIALS

Table of Contents

Supplementary Tables 2-22

Supplementary Table 1 2-3
Supplementary Table 2 4-5
Supplementary Table 3 6-11
Supplementary Table 4 12
Supplementary Table 5 13
Supplementary Table 6 14
Supplementary Table 7 15
Supplementary Table 8 16-18
Supplementary Table 9 19
Supplementary Table 10 20
Supplementary Table 11 21
Supplementary Table 12 22

Supplementary Figures 23-29

Supplementary Figure 1 23
Supplementary Figure 2 24
Supplementary Figure 3 25
Supplementary Figure 4 26
Supplementary Figure 5 27
Supplementary Figure 6 28
Supplementary Figure 7 29

Supplementary Table 1. The International Classification of Diseases for Oncology Third Edition (ICD-O-3) cancer site codes were used to identify cancer diagnoses. The Surveillance, Epidemiology, and End Results (SEER) Cause-of-death codes were used to determine cancer-specific survival outcomes.

Cancer	Site	SEER Cause-of-death
Bladder	C670, C671, C672, C673, C674, C675, C676, C677, C678, C679	C67, 188, 584, 585, 586, 593, 599, N17, N18, N19, N28, N39, N40
Brain	C710, C711, C712, C713, C714, C717, C718, C719, C720, C721, C722, C723, C724, C725	C70, C71, C72, 191, 192
Breast	C500, C501, C502, C503, C504, C505, C506, C508, C509	C50, 174, 175, 611, N61, N62, N63, N64
Colon/rectum	C180, C182, C183, C184, C185, C186, C187, C188, C189, C199, C209	153, 1590, C18, C260, 1540, 1541, 1542, 1543, 1548, 1890, 1891, C19, C20, C21, C64, C65, 530, 531, 532, 533, 534, 535, 536, 537, 538, 539, 540, 541, 542, 543, 556, 557, 558, 559, 560, 561, 562, 567, 568, 569, 578, 042, 042, 044, 530, 531, 532, 533, 534, 535, 536, 537, 556, 557, 558, 559, 560, 561, 562, 569, 578, K20, K21, K22, K23, K24, K25, K26, K27, K28, K29, K30, K31, K35, K36, K37, K38, K51, K52, K53, K54, K55, K56, K57, K62, K63, K65, K66, K92
Esophagus	C150, C151, C152, C153, C154, C155, C158, C159	150, C15, 530, 531, 532, 533, 534, 535, 536, 537, 556, 557, 558, 559, 560, 561, 562, 578, K20, K21, K22, K23, K24, K25, K26, K27, K28, K29, K30, K31, K51, K52, K53, K54, K55, K56, K57, K92
Kidney	C649, C659	C64, C65, 1890, 1891, 584, 585, 586, 593, 599, N17, N18, N19, N28, N39, N40
Leukemia	C420, C421	2040, 2041, 2024, 2042, 2048, 2049, 2050, 2070, 2072, 2060, 2051, 2052, 2053, 2058, 2059, 2061, 2062, 2068, 2069, 2080, 2031, 2071, 2078, 2081, 2082, 2088, 2089, C910, C911, C912, C913, C914, C916, C917, C918, C919, C920, C924, C925, C926, C928, C940, C942, C930, C921, C922, C923, C927, C929, C931, C932, C933, C937, C939, C944, C945, C950, C901, C915, C941, C943, C947, C951, C952, C957, C959
Liver	C220, C221	1550, 1552, C220, C222, C223, C224, C227, C229, 530, 531, 532, 533, 534, 535, 536, 537, 556, 557, 558, 559, 560, 561, 562, 570, 571, 572, 573, 578, K20, K21, K22, K23, K24, K25, K26, K27, K28, K29, K30, K31, K51, K52, K53, K54, K55, K56, K57, K70, K71, K72, K73, K74, K75, K76, K92
Lung	C340, C341, C342, C343, C348, C349	C34, 1622, 1623, 1624, 1625, 1628, 1629
Lymphoma	C770, C771, C772, C773, C774, C775, C778, C779	C82, C83, C84, C85, C86, C963, 0420, 0421, 0429, 043, 044, B20, B22, B23, B24
Melanoma	C440, C441, C442, C443, C444, C445, C446, C447,	C43, 172

	C448, C449, C510, C511, C512, C513, C514, C515, C516, C517, C518, C519, C600, C601, C602, C603, C604, C605, C606, C607, C608, C609, C632	
Ovarian	C569	1830, C56, 614, 615, 616, 617, 618, 619, 620, 621, N71, N72, N73, N74, N75, N76, N77, N78, N79, N80, N81, N82, N83, N84, N85
Pancreatic	C250, C251, C252, C253, C254, C257, C258, C259	C25, 157, 530, 531, 532, 533, 534, 535, 536, 537, 556, 557, 558, 559, 560, 561, 562, 577, 578, K20, K21, K22, K23, K24, K25, K26, K27, K28, K29, K30, K31, K51, K52, K53, K54, K55, K56, K57, K80, K81, K82, K83, K85, K86, K92
Prostate	C619	C61, 185, 600, 601, 602, 603, 604, 605, 606, 607, 608, N40, N41, N42, N43, N44, N45, N46, N47, N48, N49, N50
Stomach	C160, C161, C162, C163, C164, C165, C166, C168, C169	C16, 151, 530, 531, 532, 533, 534, 535, 536, 537, 556, 557, 558, 559, 560, 561, 562, 578, K20, K21, K22, K23, K24, K25, K26, K27, K28, K29, K30, K31, K51, K52, K53, K54, K55, K56, K57, K92
Uterine	C540, C541, C542, C543, C548, C549, C559	182, C54, 179, C55, 614, 615, 616, 617, 618, 619, 620, 621, N71, N72, N73, N74, N75, N76, N77, N78, N79, N80, N81, N82, N83, N84, N85

Supplementary Table 2. Comparison of subjects with missing vs. available home prices. P-values are from chi-squared tests for categorical variables and Mann-Whitney U tests for continuous variables.

Median (IQR)		Home Price Missing (n = 379,500)	Home Price Available (n = 667,291)	P-value
Year of Diagnosis		2006 (2001, 2012)	2007 (2001, 2012)	<0.001
Age at Diagnosis, years		68 (58, 77)	67 (57, 75)	<0.001
Median Home Value from ACS		123,900 (83,600, 167,700)	138,400 (95,100, 185,000)	<0.001
ADI, units		99.0 (88.5, 112.9)	93.1 (83.0, 104.6)	<0.001
Proportion of homes occupied by owner		0.7 (0.4, 0.8)	0.8 (0.6, 0.9)	<0.001
Follow up, days (to death or censoring)		960 (239, 2663)	1290 (338, 3142)	<0.001
**n (%)**				
Missing ACS Median Home Value		420 (0.11)	14 (0.002)	
Missing ADI		420 (0.11)	14 (0.002)	
Missing Proportion Owner occupied		420 (0.11)	14 (0.002)	
Block Group Contains an Urban Area		249,919 (66)	469,751 (70)	<0.001
Cancer				<0.001
	Bladder	18,861 (5)	33,445 (5)	
	Brain	5,007 (1.3)	9,375 (1.4)	
	Breast	70,412 (19)	134,719 (20)	
	Colon	48,690 (13)	77,338 (12)	
	Esophagus	5,492 (1.4)	8,089 (1.2)	
	Kidney	13,146 (3.5)	23,614 (3.5)	
	Leukemia	18,030 (4.8)	30,222 (4.5)	
	Liver	5,426 (1.4)	7,070 (1.1)	
	Lung	73,474 (19)	106,695 (16)	
	Lymphoma	13,444 (3.5)	24,189 (3.6)	
	Melanoma	23,779 (6.3)	52,409 (7.9)	
	Ovarian	6,084 (1.6)	10,725 (1.6)	
	Pancreatic	10,358 (2.7)	17,343 (2.6)	
	Prostate	46,977 (12)	97,192 (15)	
	Stomach	6,811 (1.8)	10,371 (1.6)	
	Uterine	13,509 (3.6)	24,495 (3.7)	

Stage at Diagnosis				<0.001
	In situ	28,919 (7.6)	61,718 (9.2)	
	Localized	148,820 (39)	284,265 (43)	
	Regional	76,956 (20)	131,267 (20)	
	Distant	89,382 (24)	140,055 (21)	
	Unstaged	35,423 (9.3)	49,986 (7.5)	
All-cause Mortality		235,176 (62)	353,838 (53)	<0.001
Cancer-specific Mortality		126,594 (33)	189,972 (28)	<0.001

ADI = Area Deprivation Index
ACS = American Community Survey

Supplementary Table 3. Demographic characteristics

	**Total cohort**	**n=667,277**
**Median (IQR)**		
Year of diagnosis		2007 (2001, 2012)
Age of diagnosis, years		67 (57, 75)
Home price, $		201500 (140900, 291800)
Area Deprivation Index, unit		93.07 (82.96, 104.58)
Follow-up, days		1290 (338, 3142)
**n (%)**		
Stage at diagnosis		
	In situ	61718 (9.25)
	Localized	284259 (42.60)
	Regional	131264 (19.67)
	Distant	140051 (20.99)
	Unstaged	49985 (7.49)
All-cause mortality		353832 (53.03)
Cancer-specific mortality		189968 (28.47)

	**Bladder cohort**	**n=33,445**
**Median (IQR)**		
Year of diagnosis		2006 (2001, 2012)
Age of diagnosis, years		72 (64, 80)
Home price, $		195300 (138900, 275500)
Area Deprivation Index, unit		93.48 (83.75, 104.26)
Follow-up, days		1416 (482, 3074)
**n (%)**		
Stage at diagnosis		
In situ		14772 (44.17)
Localized		12760 (38.15)
Regional		2705 (8.09)
Distant		1168 (3.49)
Unstaged		2040 (6.10)
All-cause mortality		19479 (58.24)
Cancer-specific mortality		6412 (19.17)

	**Brain and nervous system cohort**	**n=9,375**
**Median (IQR)**		
Year of diagnosis		2007 (2001, 2012)
Age of diagnosis, years		61 (49, 72)
Home price, $		216400 (150900, 312800)
Area Deprivation Index, unit		91.98 (81.89, 102.69)
Follow-up, days		311 (106, 824)
**n (%)**		
Stage at diagnosis		
In situ		NA
Localized		6853 (73.10)
Regional		811 (8.65)
Distant		196 (2.09)
Unstaged		1515 (16.16)
All-cause mortality		7551 (80.54)

Cancer-specific mortality	5726 (61.08)

**Breast cohort**	**n=134,714**
**Median (IQR)**	
Year of diagnosis	2007 (2001, 2012)
Age of diagnosis, years	62 (51, 72)
Home price, $	211500 (147100, 310400)
Area Deprivation Index, unit	91.92 (81.61, 103.25)
Follow-up, days	2360 (1023, 4391)
**n (%)**	
Stage at diagnosis	
In situ	24472 (18.17)
Localized	69046 (51.25)
Regional	32158 (23.87)
Distant	5621 (4.17)
Unstaged	3417 (2.54)
All-cause mortality	40929 (30.38)
Cancer-specific mortality	16503 (12.25%)

**Colon and rectum cohort**	**n=77,338**
**Median (IQR)**	
Year of diagnosis	2005 (2000, 2011)
Age of diagnosis, years	70 (60, 79)
Home price, $	188000 (133125, 269100)
Area Deprivation Index, unit	94.71 (84.77, 106.03)
Follow-up, days	1338 (417, 3147)
**n (%)**	
Stage at diagnosis	
In situ	4650 (6.01)
Localized	26275 (33.97)
Regional	27653 (35.76)
Distant	12916 (16.70)
Unstaged	5844 (7.56)
All-cause mortality	46884 (60.62)
Cancer-specific mortality	23840 (30.83)

**Esophagus cohort**	**n=8,089**
**Median (IQR)**	
Year of diagnosis	2007 (2001, 2012)
Age of diagnosis, years	68 (59, 76)
Home price, $	187800 (132000, 268400)
Area Deprivation Index, unit	94.98 (85.18, 106.29)
Follow-up, days	291 (114, 733)
**n (%)**	
Stage at diagnosis	
In situ	147 (1.82)
Localized	1560 (19.29)
Regional	2281 (28.20)
Distant	2588 (31.99)
Unstaged	1513 (18.70)
All-cause mortality	6840 (84.56)
Cancer-specific mortality	5195 (64.22)

Kidney cohort	n=23,614
**Median (IQR)**	
Year of diagnosis	2008 (2003, 2013)
Age of diagnosis, years	65 (55, 74)
Home price, $	199150 (139200, 285975)
Area Deprivation Index, unit	93.69 (83.62, 105.26)
Follow-up, days	1364 (403, 2943)
**n (%)**	
Stage at diagnosis	
In situ	NA
Localized	14794 (62.65)
Regional	3964 (16.79)
Distant	3417 (14.47)
Unstaged	1439 (6.09)
All-cause mortality	10936 (46.31)
Cancer-specific mortality	5444 (23.05)

Leukemia cohort	n=30,221
**Median (IQR)**	
Year of diagnosis	2008 (2002, 2012)
Age of diagnosis, years	69 (59, 78)
Home price, $	203000 (143000, 291100)
Area Deprivation Index, unit	92.87 (82.83, 104.30)
Follow-up, days	730 (185, 1930)
**n (%)**	
Stage at diagnosis	
In situ	NA
Localized	NA
Regional	NA
Distant	30221 (100.00)
Unstaged	NA
All-cause mortality	20353 (67.35)
Cancer-specific mortality	7856 (26.00)

Liver and bile duct cohort	n=7,070
**Median (IQR)**	
Year of diagnosis	2009 (2003, 2013)
Age of diagnosis, years	66 (57, 76)
Home price, $	185000 (128500, 271175)
Area Deprivation Index, unit	95.96 (85.27, 109.26)
Follow-up, days	184 (57, 555)
**n (%)**	
Stage at diagnosis	
In situ	NA
Localized	2491 (35.23)
Regional	1641 (22.96)
Distant	1315 (18.60)
Unstaged	1623 (22.96)
All-cause mortality	5875 (83.10)
Cancer-specific mortality	3483 (49.26)

Lung cohort	n=106,693
**Median (IQR)**	
Year of diagnosis	2006 (2001, 2012)
Age of diagnosis, years	70 (62, 77)

Home price, $	177500 (126100, 250600)
Area Deprivation Index, unit	96.18 (86.26, 108.32)
Follow-up, days	280 (90, 746)
**n (%)**	
Stage at diagnosis	
In situ	NA
Localized	20850 (19.54)
Regional	27019 (25.32)
Distant	47094 (44.14)
Unstaged	11730 (10.99)
All-cause mortality	91489 (85.75)
Cancer-specific mortality	71668 (67.17)

**Non-Hodgkin Lymphoma cohort**	**n=24,187**
**Median (IQR)**	
Year of diagnosis	2007 (2001, 2012)
Age of diagnosis, years	65 (52, 75)
Home price, $	206600 (144200, 296900)
Area Deprivation Index, unit	92.62 (82.62, 103.70)
Follow-up, days	1378 (361, 3180)
**n (%)**	
Stage at diagnosis	
In situ	NA
Localized	4137 (17.10)
Regional	4779 (19.76)
Distant	12050 (49.82)
Unstaged	3221 (13.32)
All-cause mortality	12443 (51.44)
Cancer-specific mortality	6143 (25.40)

**Melanoma cohort**	**n=52,408**
**Median (IQR)**	
Year of diagnosis	2009 (2003, 2013)
Age of diagnosis, years	63 (50, 74)
Home price, $	241900 (168700, 351600)
Area Deprivation Index, unit	89.06 (78.75, 99.31)
Follow-up, days	1975 (785, 3774)
**n (%)**	
Stage at diagnosis	
In situ	17677 (33.73)
Localized	25176 (48.04)
Regional	3891 (7.42)
Distant	1626 (3.10)
Unstaged	4038 (7.70)
All-cause mortality	14727 (28.10)
Cancer-specific mortality	3566 (6.80)

**Ovarian cohort**	**n=10,724**
**Median (IQR)**	
Year of diagnosis	2005 (2000, 2011)
Age of diagnosis, years	63 (52, 73)
Home price, $	203550 (142100, 292900)
Area Deprivation Index, unit	92.70 (82.80, 103.99)
Follow-up, days	1060 (366, 2577)
**n (%)**	

Stage at diagnosis	
In situ	NA
Localized	2063 (19.24)
Regional	2305 (21.49)
Distant	5457 (50.89)
Unstaged	899 (8.38)
All-cause mortality	6761 (63.05)
Cancer-specific mortality	5074 (47.31)

**Pancreatic cohort**	**n=17,343**
**Median (IQR)**	
Year of diagnosis	2008 (2002, 2012)
Age of diagnosis, years	70 (61, 78)
Home price, $	199800 (140500, 285550)
Area Deprivation Index, unit	93.62 (83.52, 105.43)
Follow-up, days	157 (55, 375)
**n (%)**	
Stage at diagnosis	
In situ	NA
Localized	1497 (8.63)
Regional	5042 (29.07)
Distant	7826 (45.12)
Unstaged	2978 (17.17)
All-cause mortality	15769 (90.92)
Cancer-specific mortality	13584 (78.33)

**Prostate cohort**	**n=97,190**
**Median (IQR)**	
Year of diagnosis	2006 (2001, 2011)
Age of diagnosis, years	67 (61, 74)
Home price, $	215300 (149600, 312000)
Area Deprivation Index, unit	91.99 (81.85, 103.33)
Follow-up, days	2474 (1153, 4202)
**n (%)**	
Stage at diagnosis	
In situ	NA
Localized	76640 (78.86)
Regional	9827 (10.11)
Distant	3911 (4.02)
Unstaged	6812 (7.01)
All-cause mortality	37428 (38.51)
Cancer-specific mortality	8372 (8.61)

**Stomach cohort**	**n=10,371**
**Median (IQR)**	
Year of diagnosis	2006 (2001, 2012)
Age of diagnosis, years	71 (61, 79)
Home price, $	188200 (130500, 269300)
Area Deprivation Index, unit	94.50 (84.46, 107.01)
Follow-up, days	346 (105, 1149)
**n (%)**	
Stage at diagnosis	
In situ	NA
Localized	2713 (26.16)
Regional	2904 (28.00)
Distant	2994 (28.87)

Unstaged	1760 (16.97)
All-cause mortality	8052 (77.64)
Cancer-specific mortality	3971 (38.29)

<u>**Uterine cohort**</u>	**n=24,495**
**Median (IQR)**	
Year of diagnosis	2008 (2001, 2012)
Age of diagnosis, years	63 (55, 71)
Home price, $	198400 (139200, 287600)
Area Deprivation Index, unit	93.35 (83.12, 104.67)
Follow-up, days	2026 (735, 4155)
**n (%)**	
Stage at diagnosis	
In situ	NA
Localized	17404 (71.05)
Regional	4284 (17.49)
Distant	1651 (6.74)
Unstaged	1156 (4.72)
All-cause mortality	8316 (33.95)
Cancer-specific mortality	3131 (12.78)

Supplementary Table 4. Hazard ratios from unadjusted Cox proportional hazards models for all-cause and cancer-specific mortality based on home price for all cancers combined and each cancer individually.

	All-cause mortality HR (95% CI)	Cancer-specific mortality HR (95% CI)
All cancers	0.869 (0.866, 0.872)	0.904 (0.900, 0.908)
Bladder	0.880 (0.867, 0.894)	0.922 (0.898, 0.946)
Brain	0.932 (0.911, 0.954)	0.954 (0.930, 0.980)
Breast	0.791 (0.783, 0.798)	0.826 (0.814, 0.839)
Colon/rectum	0.883 (0.875, 0.892)	0.917 (0.905, 0.929)
Esophagus	0.906 (0.885, 0.929)	0.931 (0.905, 0.957)
Kidney	0.897 (0.881, 0.914)	0.934 (0.909, 0.959)
Leukemia	0.879 (0.867, 0.891)	0.903 (0.883, 0.923)
Liver	0.920 (0.898, 0.943)	0.868 (0.841, 0.896)
Lung	0.933 (0.927, 0.939)	0.938 (0.931, 0.945)
Lymphoma	0.849 (0.834, 0.865)	0.875 (0.853, 0.898)
Melanoma	0.754 (0.742, 0.767)	0.788 (0.762, 0.815)
Ovarian	0.889 (0.868, 0.910)	0.902 (0.878, 0.928)
Pancreatic	0.896 (0.882, 0.910)	0.905 (0.890, 0.921)
Prostate	0.797 (0.789, 0.805)	0.807 (0.790, 0.825)
Stomach	0.920 (0.900, 0.940)	0.912 (0.884, 0.941)
Uterine	0.845 (0.827, 0.864)	0.880 (0.849, 0.912)

Supplementary Table 5. Hazard ratios from adjusted Cox proportional hazards models for all-cause and cancer-specific mortality based on home price, age at diagnosis, and stage at diagnosis for all cancers combined and each cancer individually.

	**All-cause mortality**	**Cancer-specific mortality**
All cancers	0.888 (0.885, 0.891)	0.915 (0.911, 0.919)
Bladder	0.898 (0.884, 0.911)	0.949 (0.925, 0.974)
Brain	0.939 (0.918, 0.961)	0.958 (0.933, 0.984)
Breast	0.878 (0.870, 0.887)	0.887 (0.873, 0.900)
Colon/rectum	0.915 (0.906, 0.923)	0.936 (0.924, 0.948)
Esophagus	0.919 (0.897, 0.942)	0.944 (0.918, 0.971)
Kidney	0.908 (0.891, 0.926)	0.945 (0.920, 0.971)
Leukemia	0.898 (0.885, 0.910)	0.920 (0.900, 0.941)
Liver	0.914 (0.892, 0.937)	0.866 (0.838, 0.894)
Lung	0.919 (0.913, 0.926)	0.927 (0.920, 0.934)
Lymphoma	0.874 (0.858, 0.891)	0.899 (0.876, 0.923)
Melanoma	0.841 (0.827, 0.855)	0.890 (0.860, 0.921)
Ovarian	0.908 (0.886, 0.930)	0.916 (0.890, 0.941)
Pancreatic	0.904 (0.890, 0.918)	0.916 (0.901, 0.931)
Prostate	0.863 (0.854, 0.872)	0.891 (0.872, 0.910)
Stomach	0.929 (0.909, 0.950)	0.925 (0.897, 0.954)
Uterine	0.872 (0.852, 0.892)	0.915 (0.882, 0.949)

Supplementary Table 6. Marginal estimated all cause survival probabilities at 5 and 10-years from a Cox proportional hazards model incorporating home price, cancer site, age at diagnosis, and ADI. Predicted probabilities for varying home values between +/- 2 standard deviations from the mean were calculated across a reference grid of covariate values and averaged. 95% percentile confidence intervals were obtained via bootstrap resampling with 1000 replicates.

	**Standard deviations above and below the mean home price**				
	**-2**	**-1**	**0**	**1**	**2**
**5-year survival, % (95% CI)**	55.7 (55.5, 56.0)	57.5 (57.3, 57.7)	59.3 (59.2, 59.4)	61.0 (60.9, 61.2)	62.7 (62.5, 63.0)
**10-year survival, % (95% CI)**	41.3 (41.0, 41.6)	43.2 (43.0, 43.4)	45.2 (45.0, 45.3)	47.1 (46.9, 47.3)	49.0 (48.7, 49.3)

Supplementary Table 7. P-values from Cox models assessing home price by ADI interaction for all cancers combined and each cancer individually. The home price-ADI interaction term describes how much the log hazard for home price changes per standard deviation increase in ADI.

	**All-cause mortality P-value**	**Cancer-specific mortality P-value**
All cancers	<0.001	<0.001
Bladder	0.78	0.24
Brain	0.83	0.28
Breast	<0.001	0.21
Colon/rectum	0.02	0.09
Esophagus	0.42	0.59
Kidney	0.03	0.05
Leukemia	<0.001	<0.001
Liver	0.05	0.14
Lung	<0.001	<0.001
Lymphoma	0.17	0.14
Melanoma	0.26	0.34
Ovarian	0.66	0.59
Pancreatic	<0.001	<0.001
Prostate	<0.001	0.26
Stomach	0.02	0.31
Uterine	0.56	0.04

Supplementary Table 8. Kaplan-Meier median all cause survival times (and 95% confidence intervals) for individual cancers stratified by quartile of home price estimates and ADI. Cells containing "NA" reflect situations where survival does not drop below 50%.

		Median survival, days (95% CI)			
		ADI Quartile 1	Quartile 2	Quartile 3	Quartile 4
**Bladder**	**Home price**	**(Least deprived)**			**(Most deprived)**
	Quartile 1	2180 (1826, 3508)	2185 (1907, 2423)	2215 (2061, 2421)	1944 (1836, 2107)
	Quartile 2	2243 (1924, 2554)	2389 (2220, 2525)	2164 (2012, 2337)	2053 (1900, 2235)
	Quartile 3	2506 (2268, 2796)	2591 (2407, 2802)	2372 (2207, 2527)	2149 (1901, 2555)
	Quartile 4	3320 (3151, 3518)	3028 (2733, 3372)	2435 (2240, 2889)	3263 (2683, 4126)
**Brain**					
	Quartile 1	519 (292, 1096)	307 (238, 444)	274 (225, 335)	304 (267, 339)
	Quartile 2	350 (246, 473)	274 (229, 320)	308 (269, 365)	307 (247, 365)
	Quartile 3	346 (305, 385)	338 (299, 375)	328 (277, 376)	316 (233, 410)
	Quartile 4	441 (417, 483)	422 (365, 495)	417 (301, 486)	446 (334, 678)
**Breast**					
	Quartile 1	5981 (5253, 7251)	5484 (5157, 5854)	4925 (4769, 5086)	4902 (4792, 5025)
	Quartile 2	6290 (6076, 6610)	5846 (5663, 6014)	5730 (5569, 5906)	5239 (5058, 5419)
	Quartile 3	6830 (6610, 7301)	6719 (6499, 6924)	6543 (6263, 6830)	5844 (5461, 6298)
	Quartile 4	NA (NA, NA)	NA (7476, NA)	NA (6743, NA)	NA (6719, NA)
**Colon and rectum**					
	Quartile 1	1900 (1422, 2316)	1804 (1707, 1954)	1826 (1729, 1942)	1712 (1641, 1790)
	Quartile 2	2115 (1933, 2376)	2011 (1920, 2162)	1994 (1894, 2127)	1846 (1741, 1993)
	Quartile 3	2442 (2285, 2610)	2232 (2132, 2351)	2113 (1965, 2268)	1822 (1636, 2058)
	Quartile 4	3060 (2853, 3213)	2538 (2369, 2747)	2517 (2133, 2839)	2170 (1841, 2582)
**Esophagus**					
	Quartile 1	345 (236, 841)	264 (204, 353)	283 (241, 345)	275 (241, 306)
	Quartile 2	337 (269, 432)	288 (259, 327)	294 (256, 338)	315 (263, 347)
	Quartile 3	343 (299, 419)	347 (301, 408)	260 (226, 303)	352 (255, 427)
	Quartile 4	367 (332, 411)	359 (310, 433)	304 (242, 415	346 (253, 546)

**Kidney**				
Quartile 1	3329 (2541, 4744)	3009 (2781, 3876)	2716 (2407, 3108)	2838 (2624, 3016)
Quartile 2	2894 (2384, 3472)	2944 (2625, 3249)	2845 (2614, 3064)	2795 (2444, 3178)
Quartile 3	3263 (2978, 3474)	3101 (2826, 3507)	3025 (2649, 3460)	2889 (2426, 3406)
Quartile 4	4458 (4192, 4917)	3988 (3516, 4343)	3658 (2997, 5012)	4751 (2527, NA)
**Leukemia**				
Quartile 1	634 (501, 1073)	824 (671, 1079)	806 (714, 929)	877 (806, 956)
Quartile 2	1059 (864, 1235)	958 (857, 1095)	859 (782, 940)	873 (747, 960)
Quartile 3	1089 (950, 1212)	1114 (991, 1250)	929 (806, 1068)	1017 (905, 1310)
Quartile 4	1849 (1719, 1991)	1354 (1196, 1543)	1223 (1006, 1549)	1099 (945, 1592)
**Liver and bile duct**				
Quartile 1	107 (85, 468)	134 (91, 217)	154 (130, 187)	181 (154, 210)
Quartile 2	181 (138, 209)	184 (153, 221)	193 (168, 240)	181 (144, 209)
Quartile 3	227 (193, 289)	197 (170, 251)	193 (164, 233)	120 (89, 213)
Quartile 4	278 (243, 312)	265 (205, 315)	201 (155, 369)	201 (106, 428)
**Lung**				
Quartile 1	297 (255, 345)	265 (246, 285)	276 (265, 290)	272 (264, 280)
Quartile 2	278 (259, 303)	291 (277, 303)	295 (283, 306)	279 (267, 290)
Quartile 3	316 (303, 333)	301 (290, 314)	306 (290, 322)	282 (263, 302)
Quartile 4	359 (348, 366)	335 (322, 350)	322 (302, 349)	293 (260, 324)
**Non-Hodgkin Lymphoma**				
Quartile 1	2664 (1995, 4908)	2228 (1746, 2712)	2262 (2024, 2593)	2024 (1835, 2212)
Quartile 2	2882 (2347, 3923)	2589 (2354, 2910)	2438 (2152, 2629)	2238 (1834, 2528)
Quartile 3	3026 (2734, 3565)	2958 (2674, 3213)	3214 (2889, 3923)	2512 (2149, 3264)
Quartile 4	4571 (4226, 4974)	3643 (3333, 4192)	3953 (3258, 4902)	3601 (2304, 5622)
**Melanoma**				
Quartile 1	5902 (4936, NA)	5391 (4742, 6070)	5034 (4762, 5475)	4492 (4225, 4691)

	Quartile 2	6079 (5453, 6731)	5716 (5493, 6089)	5221 (4969, 5613)	5174 (4835, 5446)
	Quartile 3	NA (6537, NA)	6919 (6561, NA)	7156 (6431, NA)	6532 (5825, 7215)
	Quartile 4	NA (NA, NA)	NA (NA, NA)	NA (6965, NA)	6554 (5896, NA)
**Ovarian**					
	Quartile 1	623 (493, 1461)	1363 (1096, 1791)	1073 (934, 1249)	1096 (990, 1239)
	Quartile 2	1492 (1332, 2086)	1442 (1183, 1656)	1301 (1191, 1588)	1131 (1028, 1314)
	Quartile 3	1650 (1402, 1975)	1538 (1366, 1789)	1668 (1338, 2126)	1242 (1028, 1485)
	Quartile 4	1916 (1787, 2180)	1673 (1459, 1937)	1664 (1427, 2373)	1631 (906, 3601)
**Pancreatic**					
	Quartile 1	178 (122, 246)	140 (116, 167)	139 (122, 155)	134 (123, 145)
	Quartile 2	163 (135, 189)	153 (138, 169)	151 (139, 163)	134 (122, 150)
	Quartile 3	174 (160, 188)	176 (160, 187)	170 (153, 186)	156 (132, 185)
	Quartile 4	226 (214, 242)	174 (156, 191)	208 (173, 234)	152 (110, 232)
**Prostate**					
	Quartile 1	4082 (3672, 4884)	4104 (3948, 4321)	4057 (3907, 4178)	4022 (3939, 4112)
	Quartile 2	4814 (4541, 5034)	4509 (4413, 4641)	4363 (4254, 4499)	4275 (4162, 4378)
	Quartile 3	5201 (5033, 5387)	5187 (5068, 5354)	4873 (4673, 4996)	4902 (4613, 5175)
	Quartile 4	6394 (6252, 6565)	6108 (5875, 6309)	5764 (5548, 6052)	5048 (4528, 5343)
**Stomach**					
	Quartile 1	292 (175, 708)	412 (258, 684)	366 (311, 417)	327 (293, 367)
	Quartile 2	410 (300, 660)	383 (334, 472)	411 (376, 457)	348 (294, 435)
	Quartile 3	353 (301, 419)	362 (311, 419)	342 (291, 393)	407 (315, 551)
	Quartile 4	481 (432, 538)	442 (381, 528)	447 (366, 599)	461 (387, 867)
**Uterine**					
	Quartile 1	5182 (4701, NA)	4998 (4277, 6143)	5362 (5091, 5890)	4554 (4325, 4845)
	Quartile 2	5821 (5173, NA)	5619 (5254, 6009)	5433 (5157, 5848)	5242 (4790, 5946)
	Quartile 3	6341 (5843, 6989)	6426 (5743, 7272)	5875 (5229, 6418)	4976 (4469, 5872)
	Quartile 4	NA (7380, NA)	6767 (6449, NA)	7436 (5764, NA)	6798 (5884, NA)

Supplementary Table 9. Hazard ratios from unadjusted Cox proportional hazards models for all-cause and cancer-specific mortality based on adjusted home price for all cancers combined and each cancer individually. Home prices were adjusted by median proportion of income spent on housing-related costs and then log transformed and z-scored/scaled.

	**All-cause mortality HR (95% CI)**	**Cancer-specific mortality HR (95% CI)**
All cancers	0.874 (0.871, 0.877)	0.908 (0.904, 0.912)
Bladder	0.882 (0.868, 0.895)	0.919 (0.895, 0.944)
Brain	0.938 (0.917, 0.960)	0.961 (0.936, 0.987)
Breast	0.798 (0.790, 0.805)	0.832 (0.819, 0.844)
Colon/rectum	0.888 (0.880, 0.897)	0.917 (0.905, 0.929)
Esophagus	0.906 (0.884, 0.928)	0.930 (0.904, 0.957)
Kidney	0.907 (0.890, 0.924)	0.937 (0.912, 0.962)
Leukemia	0.884 (0.872, 0.896)	0.907 (0.887, 0.928)
Liver	0.919 (0.896, 0.942)	0.867 (0.839, 0.895)
Lung	0.937 (0.931, 0.943)	0.941 (0.934, 0.948)
Lymphoma	0.858 (0.843, 0.874)	0.883 (0.860, 0.906)
Melanoma	0.760 (0.747, 0.772)	0.804 (0.777, 0.831)
Ovarian	0.894 (0.872, 0.916)	0.911 (0.886, 0.937)
Pancreatic	0.904 (0.890, 0.918)	0.913 (0.897, 0.928)
Prostate	0.809 (0.801, 0.817)	0.814 (0.797, 0.831)
Stomach	0.925 (0.905, 0.945)	0.909 (0.881, 0.938)
Uterine	0.844 (0.825, 0.863)	0.868 (0.836, 0.900)

Supplementary Table 10. Hazard ratios from adjusted Cox proportional hazards models for all-cause and cancer-specific mortality based on age at diagnosis, stage at diagnosis, and adjusted home price for all cancers combined and each cancer individually. Home prices were adjusted by median proportion of income spent on housing-related costs and then log transformed and z-scored/scaled.

	**All-cause mortality HR (95% CI)**	**Cancer-specific mortality HR (95% CI)**
All cancers	0.892 (0.889, 0.895)	0.917 (0.913, 0.922)
Bladder	0.899 (0.885, 0.913)	0.943 (0.918, 0.968)
Brain	0.950 (0.928, 0.973)	0.970 (0.944, 0.996)
Breast	0.882 (0.873, 0.890)	0.889 (0.876, 0.903)
Colon/rectum	0.917 (0.908, 0.925)	0.934 (0.921, 0.946)
Esophagus	0.915 (0.892, 0.938)	0.940 (0.913, 0.967)
Kidney	0.915 (0.897, 0.933)	0.944 (0.918, 0.970)
Leukemia	0.903 (0.891, 0.916)	0.924 (0.903, 0.945)
Liver	0.907 (0.884, 0.930)	0.860 (0.833, 0.889)
Lung	0.924 (0.918, 0.930)	0.931 (0.924, 0.938)
Lymphoma	0.881 (0.864, 0.897)	0.903 (0.879, 0.927)
Melanoma	0.846 (0.832, 0.861)	0.908 (0.877, 0.940)
Ovarian	0.910 (0.888, 0.933)	0.922 (0.896, 0.949)
Pancreatic	0.913 (0.899, 0.928)	0.923 (0.908, 0.939)
Prostate	0.869 (0.860, 0.878)	0.892 (0.873, 0.911)
Stomach	0.926 (0.905, 0.947)	0.914 (0.885, 0.943)
Uterine	0.873 (0.853, 0.893)	0.904 (0.871, 0.938)

Supplementary Table 11. Hazard ratios from adjusted Cox proportional hazards models for all-cause and cancer-specific mortality based on age at diagnosis, stage at diagnosis, ADI, and adjusted home price for all cancers combined and each cancer individually. Home prices were adjusted by median proportion of income spent on housing-related costs and then log transformed and z-scored/scaled.

	All-cause mortality HR (95% CI)	Cancer-specific mortality HR (95% CI)
All cancers	0.928 (0.924, 0.933)	0.951 (0.945, 0.957)
Bladder	0.925 (0.906, 0.944)	0.958 (0.925, 0.992)
Brain	0.959 (0.929, 0.991)	0.980 (0.944, 1.016)
Breast	0.934 (0.921, 0.947)	0.949 (0.929, 0.969)
Colon/rectum	0.947 (0.935, 0.959)	0.966 (0.949, 0.984)
Esophagus	0.939 (0.908, 0.971)	0.964 (0.928, 1.002)
Kidney	0.955 (0.930, 0.980)	0.982 (0.946, 1.019)
Leukemia	0.935 (0.917, 0.953)	0.947 (0.919, 0.977)
Liver	0.945 (0.912, 0.979)	0.893 (0.853, 0.935)
Lung	0.949 (0.940, 0.958)	0.954 (0.944, 0.964)
Lymphoma	0.923 (0.900, 0.946)	0.937 (0.904, 0.971)
Melanoma	0.889 (0.869, 0.910)	0.954 (0.911, 0.999)
Ovarian	0.954 (0.922, 0.987)	0.964 (0.927, 1.003)
Pancreatic	0.946 (0.926, 0.967)	0.958 (0.936, 0.981)
Prostate	0.898 (0.885, 0.911)	0.930 (0.903, 0.959)
Stomach	0.941 (0.913, 0.971)	0.947 (0.906, 0.989)
Uterine	0.925 (0.897, 0.954)	0.961 (0.915, 1.010)

Supplementary Table 12. P-values from Cox models assessing adjusted home price by ADI interaction for all cancers combined and each cancer individually. Home prices were adjusted by median proportion of income spent on housing-related costs and then log transformed and z-scored/scaled. The home price-ADI interaction term describes how much the log hazard for home price changes per standard deviation increase in ADI.

	**All-cause mortality**	**Cancer-specific mortality**
	**P-value**	**P-value**
All cancers	<0.001	<0.001
Bladder	0.58	0.69
Brain	0.59	0.15
Breast	<0.001	0.22
Colon/rectum	0.02	0.09
Esophagus	0.52	0.77
Kidney	0.001	0.01
Leukemia	<0.001	<0.001
Liver	0.39	0.62
Lung	<0.001	<0.001
Lymphoma	0.01	0.03
Melanoma	0.80	0.11
Ovarian	0.83	0.34
Pancreatic	<0.001	0.003
Prostate	<0.001	0.33
Stomach	0.10	0.67
Uterine	0.25	0.002

Supplementary Figure 1. Directed acyclic graph demonstrating the assumed impact of factors on survival following cancer diagnosis.

Figure legend: Conceptual model of the influence of income on survival following cancer diagnosis. Income (using home prices as a proxy) is assumed to primarily influence survival through the quality of care available to the patient. Quality of care is thought to influence the age at which patients are diagnosed and subsequently affect the stage at diagnosis. Quality of care is also assumed to directly influence survival via access to more effective treatments. Additionally, year of diagnosis may affect the quality of care available. Area deprivation is assumed to influence quality of care available and has a complex bidirectional relationship with income, as demonstrated by the dashed line between them.

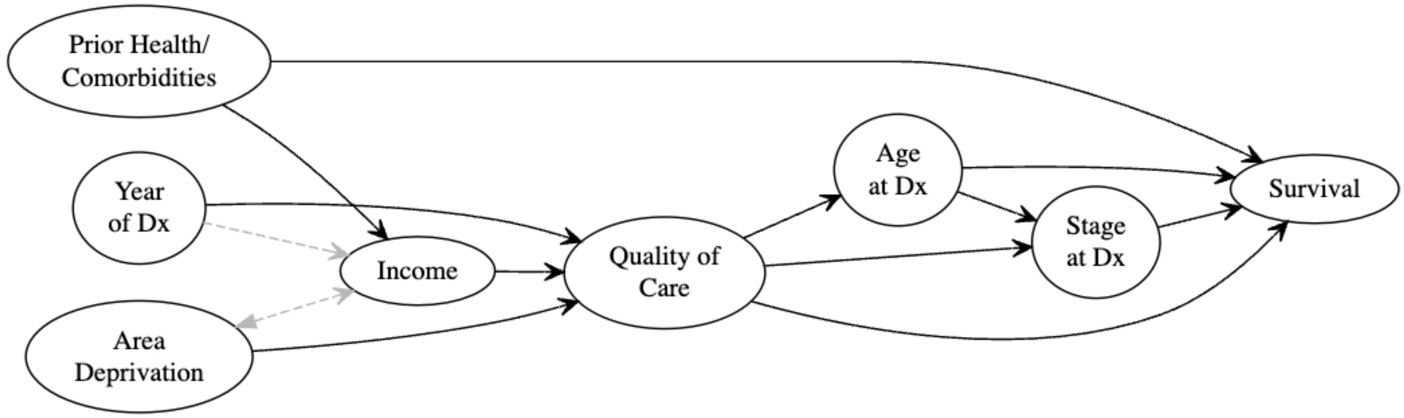

Supplementary Figure 2. Relationship between hazard of mortality and home price stratified by cancer stage at diagnosis

Figure legend: Adjusted Cox models assessing the relationship between hazard of all-cause (A) and cancer-specific (B) mortality and home price stratified by stage at diagnosis for all cancers combined and each cancer individually. Home prices were log transformed and z-scored/scaled. Hazard ratios are given at the average age at diagnosis and have been rescaled to make home price=0 and stage=localized the referent. Outer borders of each line represent 95% confidence interval bands.

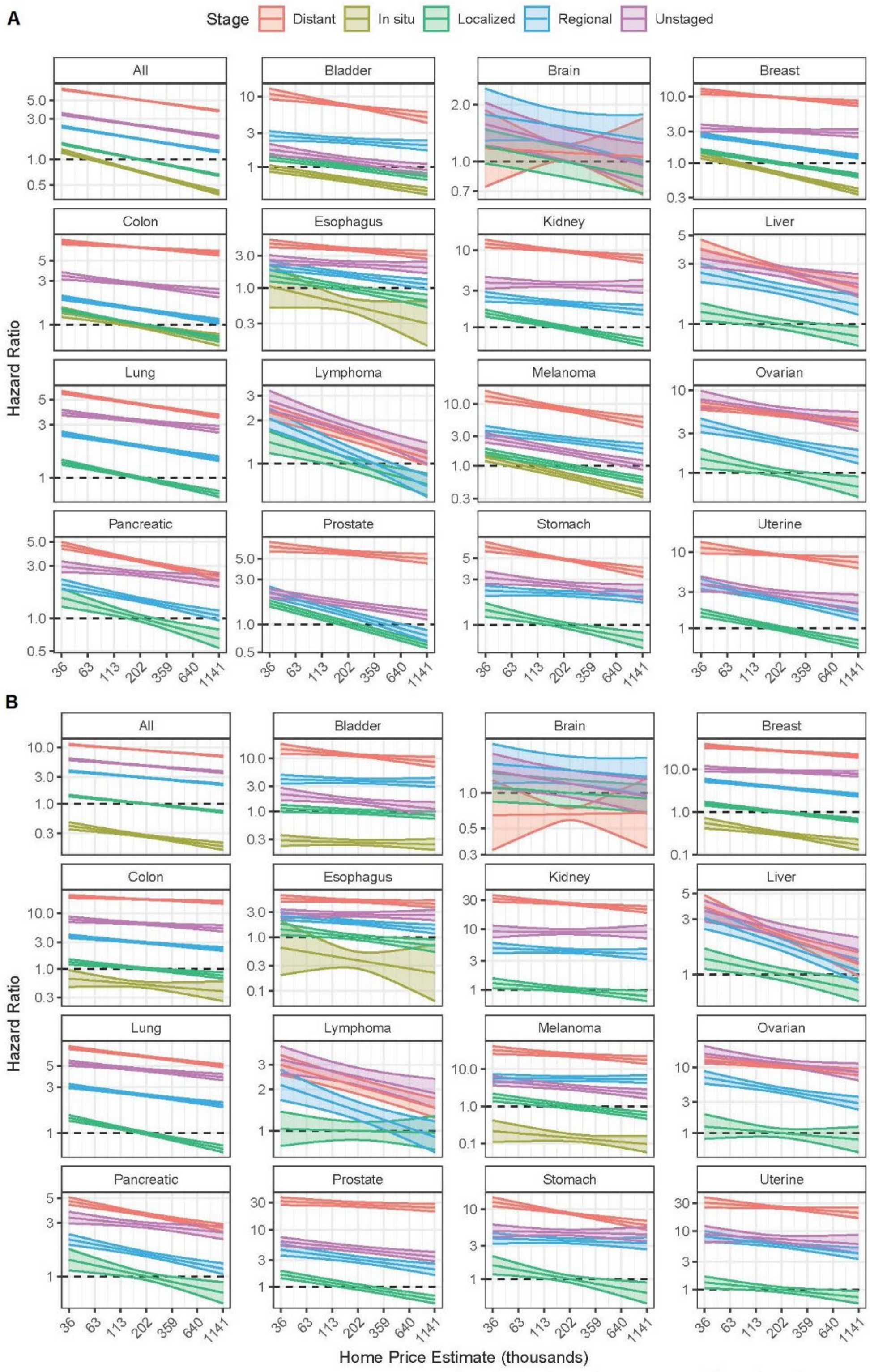

Supplementary Figure 3. Comparison of Cox models utilizing home price or area deprivation

Figure legend: Comparing hazard ratios of all-cause (A) and cancer-specific (B) mortality for all cancers combined and each cancer individually according to home price, Area Deprivation Index (ADI), or home price and ADI, with or without adjustment for age and stage at diagnosis. Cox models for 'All' cancers are adjusted for cancer sites. Hazard ratios for ADI have been flipped. Bands represent 95% confidence intervals.

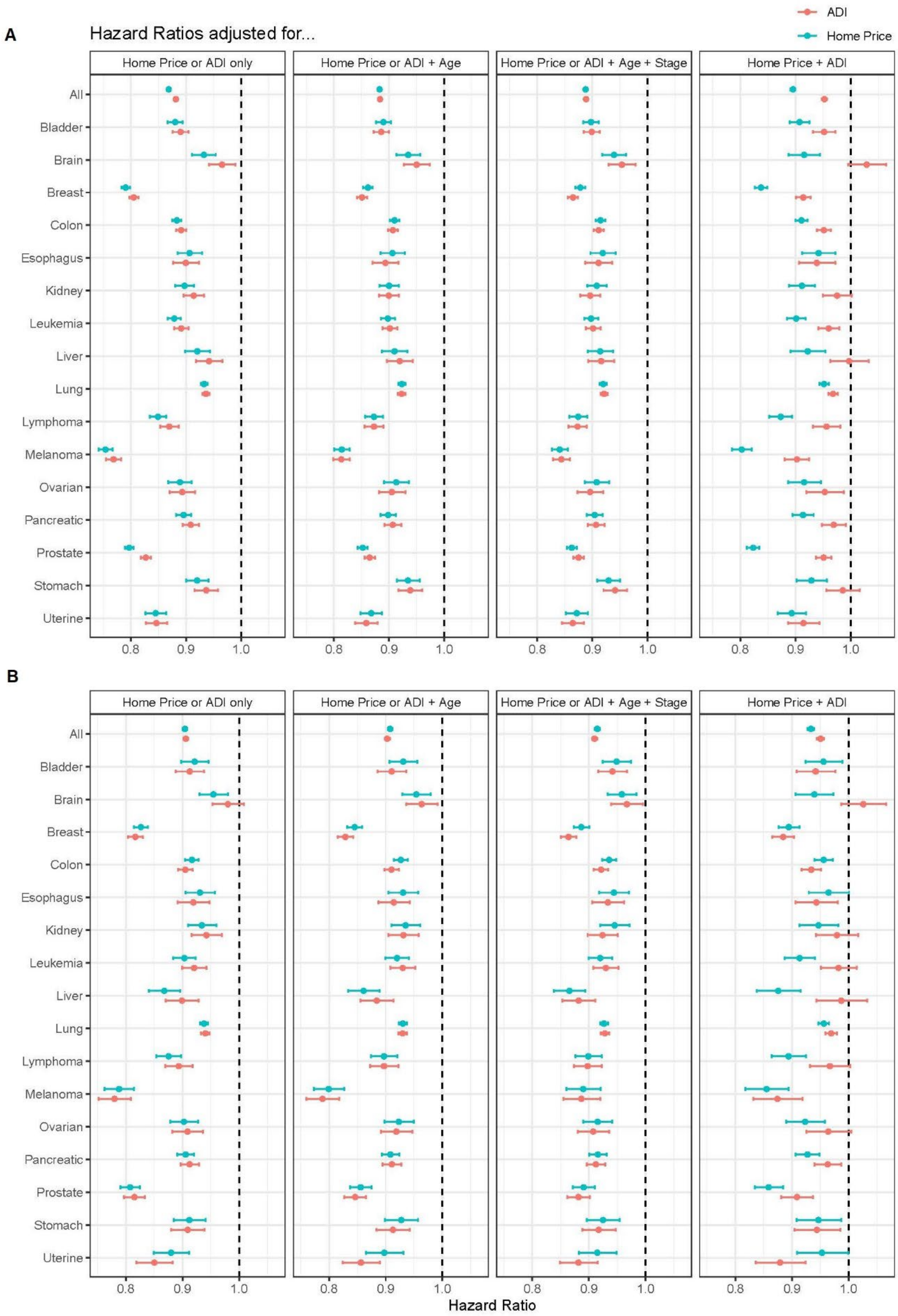

Supplementary Figure 4. Effect of area deprivation and home price on hazard of mortality for all cancers

Figure legend: Estimated hazard ratios from Cox models allowing for an interaction between home price and Area Deprivation Index (ADI) for all-cause (A) and cancer-specific (B) mortality for all cancers combined and each cancer individually. HRs are plotted for ADI values two standard deviations above (blue) or below (green) the mean (red). Cox models were constructed with ADI as a continuous (linear) predictor along with home price, age at diagnosis, and stage at diagnosis. Hazard ratios are given at the average age and localized stage at diagnosis and have been rescaled to make home price=0 and ADI=0 the referent. Outer borders of each line represent 95% confidence interval bands.

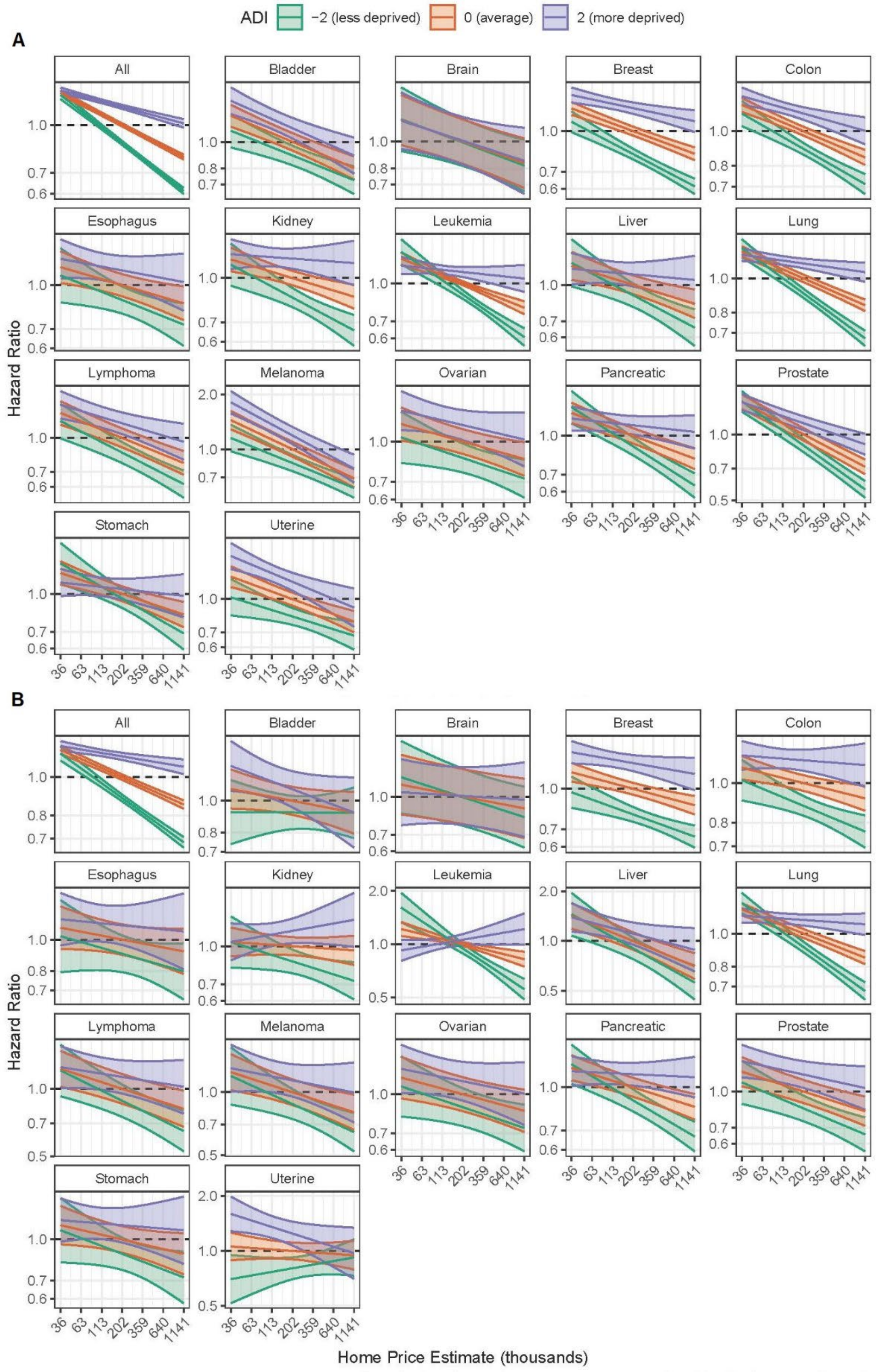

Supplementary Figure 5. Relationship between hazard of mortality and home price stratified by earlier vs. later years of diagnosis

Figure legend: Adjusted Cox models assessing the relationship between hazard of all-cause (A) and cancer-specific (B) mortality and home price for cancers stratified by earlier (1996-2005) or later (2006-2016) years of diagnosis. Hazard ratios are given at the average age and localized stage at diagnosis and have been rescaled to make home price=0 the referent. Outer borders of each line represent 95% confidence interval bands.

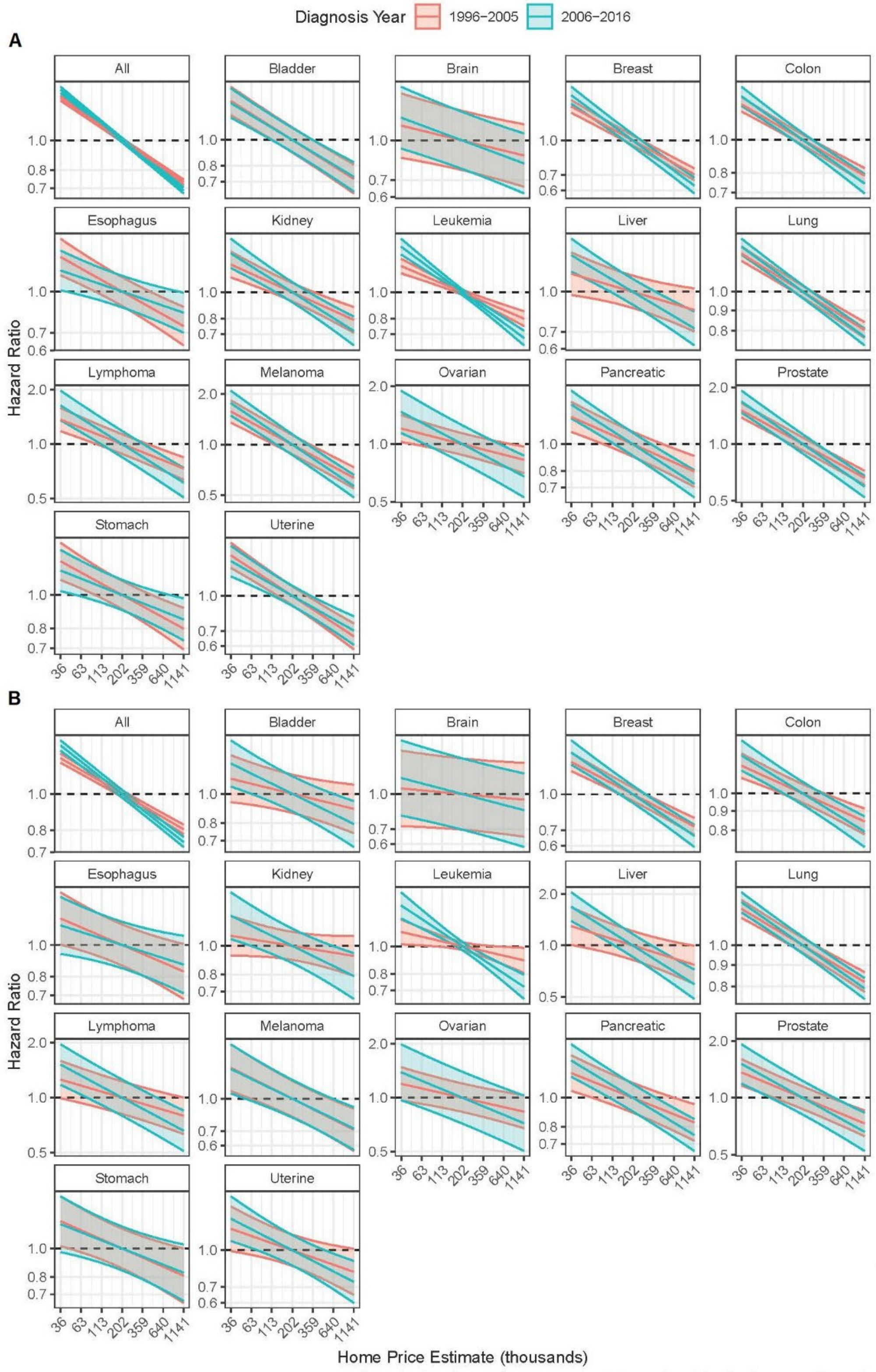

Supplementary Figure 6. Overall relationship between hazard of mortality and adjusted home price

Figure legend: Unadjusted Cox models assessing the relationship between hazard of all-cause (A) and cancer-specific (B) mortality and adjusted home prices for cancers of all stages combined. Home prices were adjusted by median proportion of income spent on housing-related costs and then log transformed and z-scored/scaled. Blue shading indicates 95% confidence interval bands.

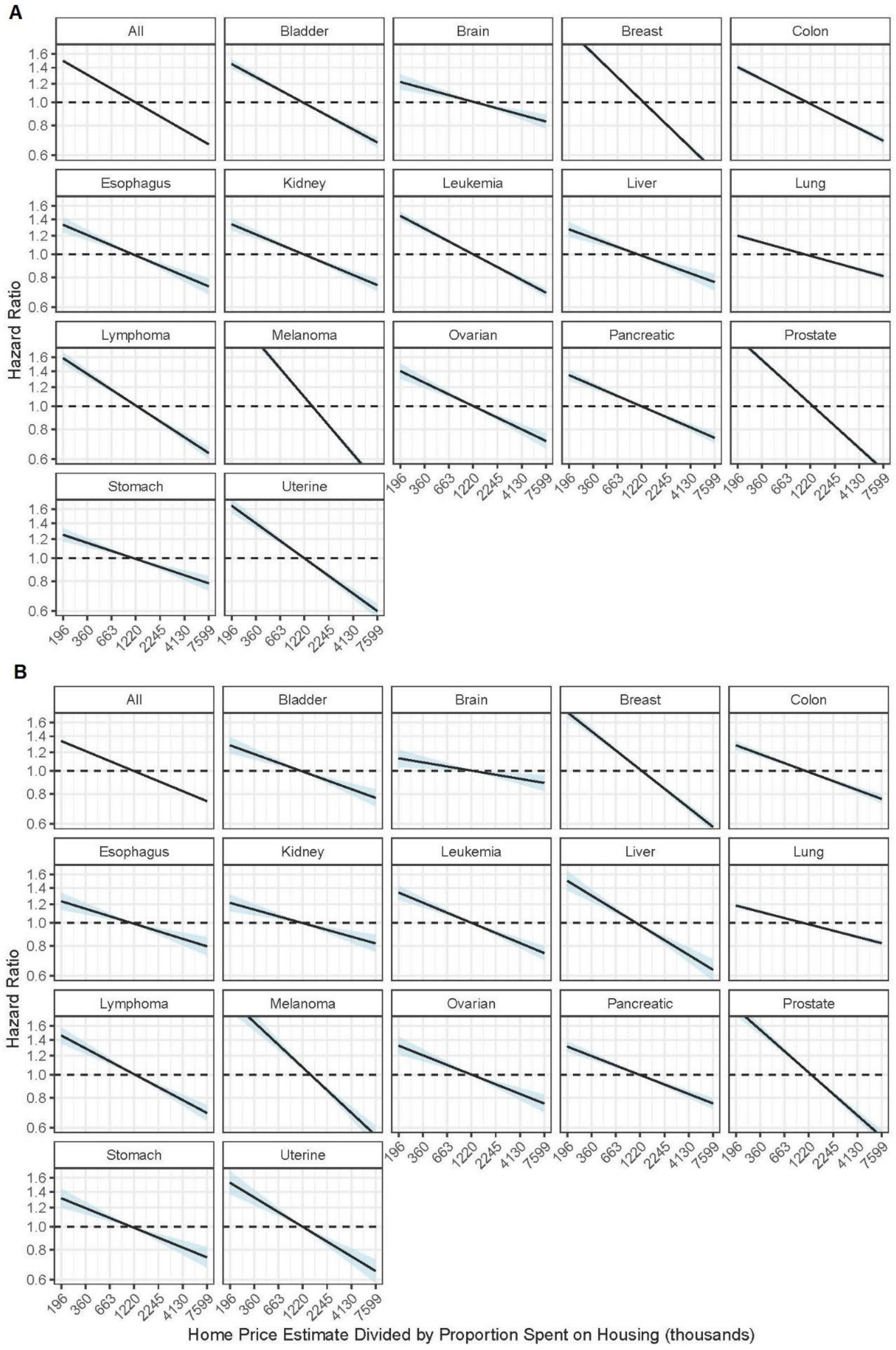

Supplementary Figure 7. Relationship between hazard of mortality and adjusted home price stratified by cancer stage at diagnosis

Figure legend: Adjusted Cox models assessing the relationship between hazard of all-cause (A) and cancer-specific (B) mortality and adjusted home price stratified by stage at diagnosis for all cancers combined and each cancer individually. Home prices were adjusted by median proportion of income spent on housing-related costs and then log transformed and z-scored/scaled. Hazard ratios are given at the average age at diagnosis and have been rescaled to make home price=0 and stage=localized the referent. Outer borders of each line represent 95% confidence interval bands.

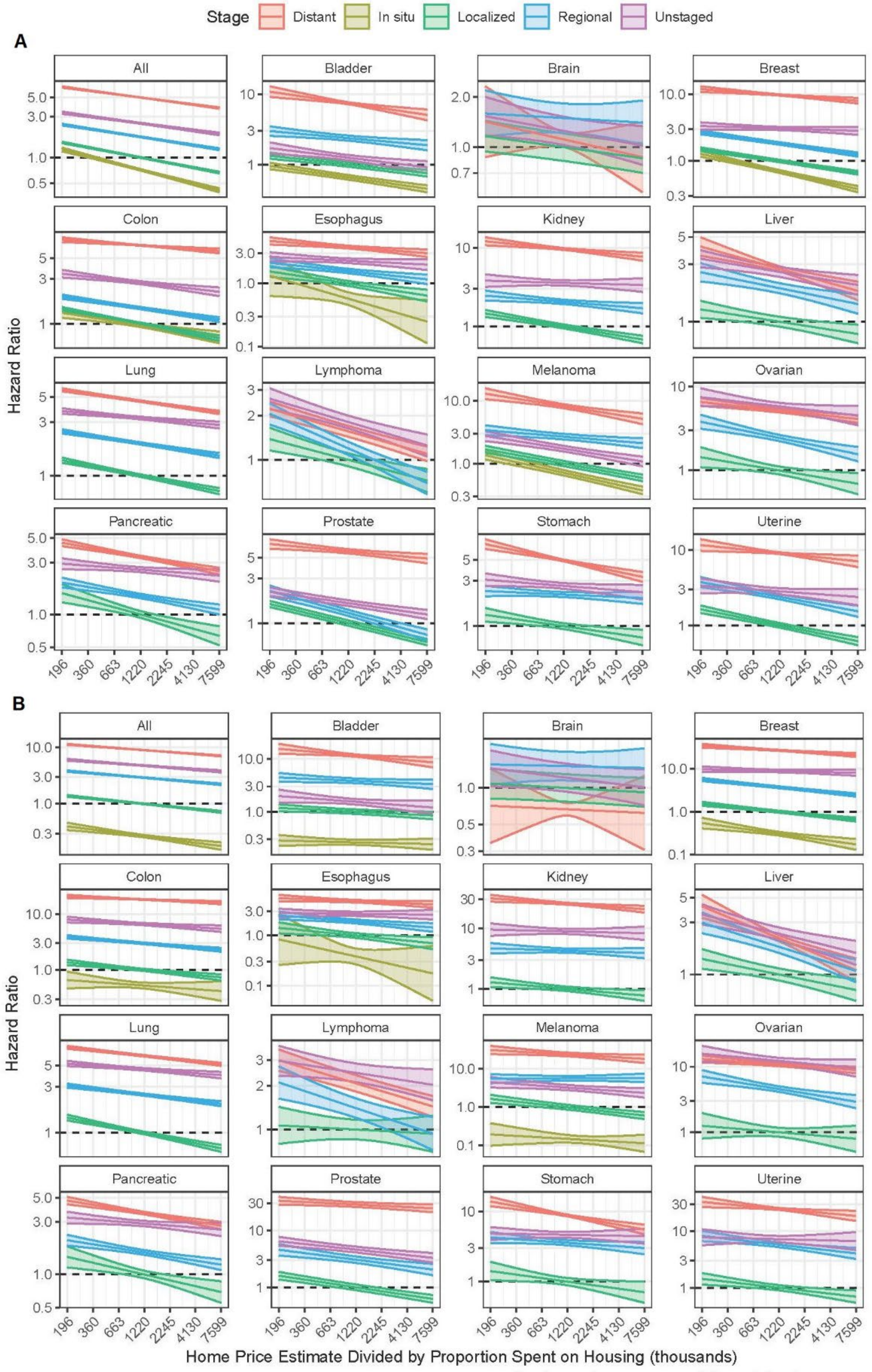

Supplement: pkad076_Supplementary_Data [file pkad076_supplementary_data.pdf]
